# The effect of micronutrient on thyroid cancer risk: a Mendelian randomization study

**DOI:** 10.3389/fnut.2024.1331172

**Published:** 2024-03-01

**Authors:** Jiali Shen, Hong Zhang, Hongzhan Jiang, Huihui Lin, Jiaxi He, Siyue Fan, Doudou Yu, Liping Yang, Hui Tang, Ende Lin, Lianghui Li, Lijuan Chen

**Affiliations:** ^1^School of Nursing, Fujian University of Traditional Chinese Medicine, Fuzhou, China; ^2^School of Clinical Medicine, Fujian Medical University, Fuzhou, China; ^3^School of Medicine, Xiamen University, Xiamen, China; ^4^Department of Nuclear Medicine, Zhongshan Hospital of Xiamen University, School of Medicine, Xiamen, China; ^5^Department of General Surgery, Zhongshan Hospital of Xiamen University, School of Medicine, Xiamen, China

**Keywords:** thyroid cancer, copper, iron, zinc, calcium, vitamin D, vitamin C, Mendelian randomization

## Abstract

**Background:**

The effect of micronutrients on thyroid cancer has been studied in observational studies, however, the cause of relationships has not yet been determined. Thyroid cancer was the subject of a Mendelian randomization (MR) analysis of micronutrients. Aimed to determine whether micronutrient intake has a causal impact on the chance of developing thyroid cancer.

**Methods:**

We used a Mendelian randomization (MR) analysis with two samples. Our circulation levels of Cu, Ir, Zn, Ca, VD, and VC were reflected by genetic variations reported from GWAS in individuals of European ancestry. For the GWAS outcome of thyroid cancer. Sensitivity studies that included MR-Egger, weighted median/mode tests, and a more open selection of variations at a genome-wide sub-significant threshold were added to our inverse-variance weighted (IVW) MR study.

**Results:**

Using the IVW approach, we did not find evidence that any of the micronutrients to thyroid cancer (Cu: odds ratio [*OR* = 0.88, *p* = 0.41]; Zn: odds ratio [*OR* = 0.87, *p* = 0.40]; Ir: odds ratio [*OR* = 1.18, *p* = 0.39]; Ca: odds ratio [*OR* = 1.12, *p* = 0.43]; VC: odds ratio [*OR* = 0.95, *p* = 0.22]; VD: odds ratio [*OR* = 0.89, *p* = 0.04]). The heterogeneity (*p* > 0.05) and pleiotropy (*p* > 0.05) testing provided confirmatory evidence for the validity of our MR estimates.

**Conclusion:**

This study does not provide evidence that supplementation with micronutrients including Cu, Ir, Zn, Ca, VD, and VC can prevent thyroid cancer.

## Introduction

The global incidence of thyroid cancer (TC) is experiencing a steady increase, primarily attributed to the heightened utilization of diagnostic imaging and surveillance. According to data obtained from the International Agency for Research on Cancer (IARC) in 2020, the Global Cancer Observatory reported a total of 586,000 newly diagnosed cases of TC worldwide, accounting for 3.0% of all newly diagnosed cancer cases (1). Furthermore, TC ranked 9th among population-based cancer incidence rates. In the United States, thyroid carcinoma is projected to rank 13th in terms of newly diagnosed tumors in 2022, with an estimated 440,000 cases (2). TC has been recognized as a cancer type with a significant financial burden on patients. Many observational studies have indicated the potential importance of micronutrients and their supplementation in preventing and alleviating cancer (3).

Copper (Cu) plays a crucial role in respiration, free-radical defense, and immune regulation, primarily attributed to its structural involvement in cuproenzymes (4). Additionally, Cu is believed to initiate angiogenesis in tumor cells (5). However, elevated levels of Cu can potentially stimulate growth, proliferation, and carcinogenesis by causing DNA damage through the action of toxic hydroxyl radicals (6).

Iron (Ir) is crucial for maintaining human health due to its involvement in oxidation–reduction reactions and its significant role in various physiological processes (7). Ongoing investigations are being conducted to explore the association between IR and thyroid cancer. Notably, thyroid cancer cells secrete hepcidin, a hormone that can suppress the expression of Ferroportin (FPN) and result in elevated intracellular Ir levels, consequently facilitating cancer cell proliferation. The majority of existing research supports the notion of a correlation between Ir and thyroid cancer (8, 9).

Zinc (Zn) is an essential trace metal with structural roles in regulatory proteins, as an enzyme cofactor, and as a signaling molecule. Zn is essential for thyroid hormone metabolism and has a potential relationship with cancer (10). Serum Zn concentrations are significantly reduced in many malignant tumors, including thyroid cancer. Zn levels in PTC and follicular carcinoma are lower than that in healthy individuals (11).

Calcium (Ca) is a mineral mainly found in bones. Intracellular Ca ions act as second messengers to regulate various physiological processes in cells, such as cell proliferation, motility, apoptosis, and other physiological processes. Some studies have found that serum Ca ions can be a protective factor for tumors (12). The thyroid gland plays a role in calcium metabolism and regulation, by secreting when needed and the thyroid stimulating hormones (TSHs) are key to regulating the metabolism of the thyroid gland to produce both T4 (thyroxine) and T3 (tri-iodothyronine) (13).

Vitamin D (VD) has a variety of physiological functions, such as regulating calcium metabolism, regulating cell growth and differentiation, regulating immunity, anti-tumor activity, and other functions (14). Some studies have analyzed and found that VD levels are significantly associated with the risk of thyroid cancer, VD may be a protective factor in the development of thyroid cancer.

Vitamin C (VC) is a necessary nutrient that regulates endothelial nitric oxide synthase and causes the neutrophil oxidative burst to maintain microcirculatory homeostasis and decrease microcirculatory pro-inflammatory signaling (15). Additionally, there is proof that consuming vitamins with antioxidant qualities, such as VC and VD, may have some positive impact on illnesses connected to the thyroid (16). It has been shown that both hyper- and hypothyroidism can affect the concentrations of the vitamins involved in the scavenging of free radicals (17).

Unfortunately, the findings are not entirely consistent and the conclusions of most studies predominantly rely on observational studies. Rigorously designed randomized controlled trials (RCT) are the gold standard for discerning causality and can effectively address potential confounders. However, due to ethical constraints, external validity limitations, difficulty in double-blind design, interference from internal and external factors, insufficient statistical efficacy, and the need for significant cost and Time. The likelihood of conducting randomized controlled trials is low.

In conclusion, the relationship between thyroid cancer and micronutrients remains a new approach to be elucidated. As the number of large-scale genome-wide association studies (GWAS) is increasing, Mendelian randomization (MR) can MR is an epidemiological and genetic research method that can utilize the natural environment to infer causality of different phenotypes (18). Research methodology that can simulate causation and more precisely measure the impact of particular factors on outcomes by using genetic diversity found in nature as a randomized experiment (19).

We decided to conduct a Mendelian Randomization (MR) analysis of these micronutrients’ potential causative effects because well-powered randomized control trials (RCT) evaluating their preventive and therapeutic potential are lacking. The well-known causal inference technique known as MR makes use of genetic variations as supporting variables (20). We want to provide fresh insight into thyroid cancer prevention by using this method to detangle the causal relationships between Cu, Ir, Zn, Ca, VC, and VD exposure and thyroid malignant tumors.

## Material and Methods

### Study design

**Figure 1:**
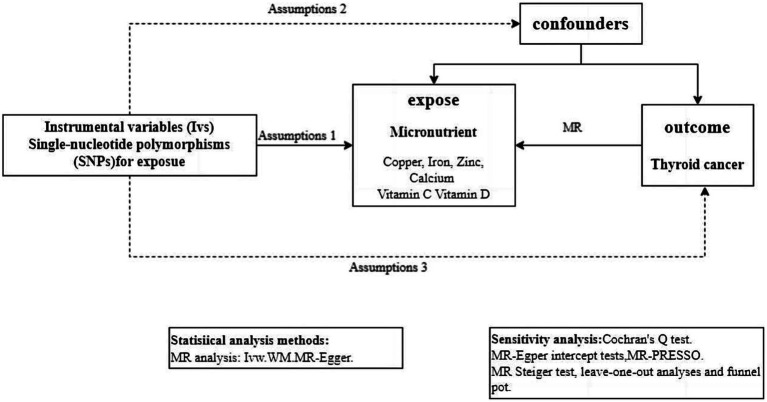
Workflow of the MR study demonstrating the link between thyroid cancer and micronutrient. IVW, inverse variance weighted; MR, Mendelian randomization; MR-PRESSO, MR pleiotropy residual sum and outlier; SNP, single-nucleotide polymorphisms.

The GWAS summary statistics about thyroid cancer and 6 micronutrients (including Cu, Ir, Zn, Ca, VC, and VD) were collected from publicly available data sources and analyzed to estimate the genetic causal relationship through MR (Figure 1).

### Determination of the instrumental variables

Three crucial aspects underlie the validity of genetic instruments: (I) Association hypothesis: There is a strong correlation between SNPs and exposure factors. (II) Independence hypothesis: SNPs are independent of confounding factors. (III) Exclusivity assumption: SNPs can only have an effect on outcomes through exposure factors (21).

### Selection of the instrumental variables

Significant SNPs were screened from the pooled GWAS data for Cu, Ir, Zn, Ca, VC, and VD (*p* < 5 × 10^−8^, Linkage disequilibrium [LD] *r*^2^ < 0.01, Genetic distance = 10,000 KB). To satisfy the first hypothesis of MR, that instrumental variables are strongly correlated with exposure, we determined the presence of weak instrumental variable bias by calculating the F statistic (F = beta^2^/se^2^) for each SNP, with *F* > 10 indicating that a weak instrumental variable is not present (22). Since the SNPs used in MR follow the principle that offspring alleles are randomly assigned from the parental generation and are highly unlikely to be influenced by the environment, we can assume that the instrumental variables are not associated with confounders and satisfy MR hypothesis II (21). In addition, this study tested for horizontal pleiotropy by MR-Egger regression model intercept and MR-PRESSO, and *p* > 0.05 indicates that there is no horizontal pleiotropy, then hypothesis three is fulfilled and the instrumental variables are associated with the outcome only through exposure (23).

### Validation of the instrumental variables

TC is a complex disease and the influence of common confounding factors on the role of both needs to be fully considered. In TC, other factors such as hormones, age, gender, smoking, and alcohol consumption may act as confounding intermediate phenotypes to further influence the development of TC. It is necessary to exclude the effects of other confounding factors. We examined whether IVs might influence the occurrence of TC through other confounding factors by validating the pleiotropy of excluding IVs.

### Data extraction

#### Data source for micronutrient

We searched the literature, in the Open GWAS (24) for genetic instruments associated with Cu, Ir, Zn, Ca, VC, and VD in populations of European ancestry. For Cu GWAS, they included 2,603 European individuals. A total of 2 SNPs related to Cu were identified (Supplementary Table S1). For Ir GWAS, they included 23,986 European individuals and identified 3 significant SNPs (Supplementary Table S2). For Ca GWAS, they included 315,153 European individuals and identified 173 significant SNPs (Supplementary Table S3). For VD GWAS, they included 496,946 European individuals and identified 118 significant SNPs (Supplementary Table S4). For VC GWAS, they included 291 European individuals and identified 68 significant SNPs (Supplementary Table S5). For Zn GWAS, they included 2,603 Europeans and a total of 2 SNPs were significant (Supplementary Table S6). In the Supplementary Material, more information on these phenotypes is available.

#### Data source for thyroid cancer

The GWAS summary data for TC were extracted from FinnGen, including 989 patients and 217,803 controls of European ancestry. The Cancer Registry data were used to identify cases of TC, according to the code C73 of the International Classification of Diseases-10.

### MR analyses

For each instrumental variable, the Wald ratio was used to evaluate the influence of exposure on the result. Then, we used the inverse variance weighted (IVW) method to combine each instrumental variable’s effect size. This method is based on the premise that instruments can only affect a result through exposure and not through any other channel (25). The weighted median and MR-Egger methods were employed to supplement IVW estimates. A stricter instrument *p*-value threshold was set if the estimations from the methods used in our investigation were inconsistent (26). All MR analysis approaches have consistent MR results, which could provide more reliable predictions in a wider range of scenarios (27). The MR analysis was combined using a random-effect model.

### Sensitivity analyses

The heterogeneity was evaluated using Cochrane’s Q value from the IVW approach, with a *p*-value <0.05 indicating statistical significance (28). The MR-Egger intercept and MR-PRESSO methods were used to detect horizontal pleiotropy (directional pleiotropy was assumed to exist if *p* < 0.05) (23), MR-PRESSO includes three components: (I) identification of horizontal pleiotropy; (II) horizontal pleiotropy correction by the elimination of outliers; (III) analyzing the causal estimates before and after the outlier correction to determine whether there are any discrepancies. A leave-one-out approach was employed to evaluate the effect of a particular SNP on the outcome of the MR analysis (28).

The MR analysis was performed by the R packages “TwoSampleMR” (version 0.5.6) and “MRPRESSO” (version 1.0) in the R software (version 4.2.0).

## Results

To investigate the causal association between Cu, Ir, Zn, Ca, VC, and VD-related SNPs and thyroid cancer, we conducted a two-sample MR study using genetic data from participants of European descent. Our analysis involved utilizing three different methods for estimating instrumental variables (IVs), including the IVW, WM, and MR-Egger. By evaluating interactions between these inputs and outcomes, our goal was to assess the validity of inferring a causal relationship (Table 1).

**Table 1:** Mendelian randomization estimates for the association between thyroid cancer and micronutrient.

Exposure	Outcome	*n* SNPs	IVW odds ratio (95% CI)	IVW *p*-value^a^	Cochrane’s *Q*	Cochrane’s *Q p*-value^a^	MR-Egger Intercept^b^	MR-Egger Intercept *p*-value^a^
VD	Thyroid cancer	115	0.89 (0.61–1.30)	0.561	115.252	0.449	−0.004	0.599
VC	Thyroid cancer	65	0.95 (0.87–1.03)	0.219	77.3101	0.123	−0.022	0.716
Ca	Thyroid cancer	189	1.12 (0.84–1.51)	0.431	187.335	0.479	−0.007	0.342
Ir	Thyroid cancer	3	1.18 (0.81–1.73)	0.385	0.161	0.923	0.014	0.869
Cu	Thyroid cancer	2	0.88 (0.65–1.20)	0.414	0.131	0.717	NA^c^	NA^c^
Zn	Thyroid cancer	2	0.87 (0.64–1.19)	0.399	0.499	0.479	NA^c^	NA^c^

a Nominal *p*-value.

b The MR-Egger intercept quantifies the effect of directional pleiotropy (*p* < 0.05, which means possible pleiotropy).

c Insufficient number of SNPs for MR-Egger analysis.

Cu, copper; Ir, iron; Ca, calcium; Zn, zinc; VD, vitamin D; VC, vitamin C; SNPs, single nucleotide polymorphisms; IVW, inverse variance weighting.

**Figure 2:**
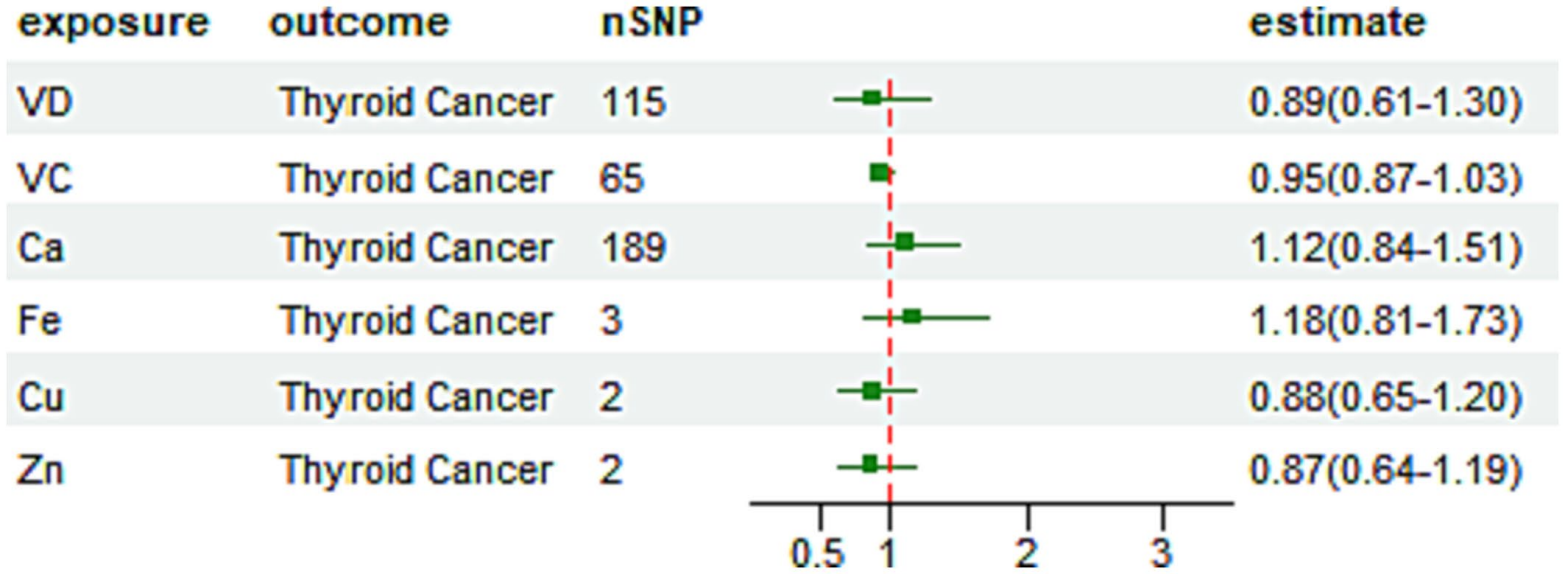
Odds ratio plot for micronutrient by IVW method.

We found little evidence in favor of the genetically predicted Cu, Ir, Zn, Ca, VC, and VD concentrations having a large effect on thyroid cancer risk (Figure 2).

In MR analysis at the *p* < 5 × 10^−8^ level, we found that the effect value of serum Cu on TC was [*OR* = 0.88, 95% CI = (0.65–1.20), *p* = 0.41], which did not support a causal effect of Cu on TC.

The IVW method of the analysis showed an effect value of serum Ir on TC [*OR* = 1.18, 95% CI = (0.81–1.73), *p* = 0.39], which did not support a causal effect of Ir on TC. The WM method also showed a similar causal effect [*OR* = 1.23, 95% CI = (0.82–1.84), *p* = 0.32]. A similar causal effect of [*OR* = 1.10, 95% CI = (0.50–2.41), *p* = 0.85] was shown in the MR-Egger analysis method. The MR-Egger model intercept term was 0.014, *p* = 0.87 and there was no evidence of any potential horizontal pleiotropy. The WM method showed a similar causal effect [*OR* = 1.23, 95% CI = (0.82–1.84), *p* = 0.32]. Scatter plots for Mendelian randomization analysis of Ir and risk of thyroid cancer are shown in Supplementary Figure S1.

The effect value of serum Zn on TC was [OR = 0.87, 95% CI = (0.64–1.19), *p* = 0.40], which did not support a causal effect of Zn on TC.

The IVW method of the analysis showed an effect value of serum Ca on TC of [*OR* = 1.12, 95% CI = (0.84–1.51), *p* = 0.43], which did not support a causal effect of Ca on TC. The WM method showed a similar causal effect [*OR* = 0.91, 95% CI = (0.51–1.62), *p* = 0.75]. And a similar causal effect was shown in the MR-Egger analysis method [OR = 1.40, 95% CI = (0.82–2.39), *p* = 0.22]. No outliers were detected by MR-PRESSO (*p* = 0.47), and with an MR-Egger model intercept term of −0.00712, *p* = 0.34, there was no evidence of any potential horizontal pleiotropy. Scatter plots for Mendelian randomization analysis of Ca and risk of thyroid cancer are shown in Supplementary Figure S2.

The IVW method of the analysis showed an effect value of VC on [*OR* = 0.95, 95% CI = (0.87–1.03), *p* = 0.22], which did not support a causal effect of VC on TC. The WM method showed a similar causal effect [*OR* = 0.94, 95% CI = (0.83–1.07), *p* = 0.40]. While in the MR-Egger analysis method, a similar causal effect of [*OR* = 0.98, 95% CI = (0.79–1.22), *p* = 0.87] was shown no outliers were detected by MR-PRESSO (*p* = 0.14), and with an MR-Egger model intercept term of −0.02202, *p* = 0.72, there was no evidence of any potential horizontal pleiotropy. Scatter plots for Mendelian randomization analysis of VC and risk of thyroid cancer are shown in Supplementary Figure S3.

The IVW method of the analysis showed an effect value of VD on TC [*OR* = 0.89, 95% CI = (0.61–1.30), *p* = 0.04], which did not support a causal effect of VD on TC. The WM method showed a similar causal effect [*OR* = 1.17, 95% CI = (0.58–2.35), *p* = 0.03]. And a similar causal effect was shown in the MR-Egger analysis method [*OR* = 1.01, 95% CI = (0.56–1.81), *p* = 0.98]. No outliers were detected by MR-PRESSO (*p* = 0.40), and with an MR-Egger model intercept term of −0.00441, *p* = 0.60, there was no evidence of any potential for horizontal multicollinearity. Scatter plots for Mendelian randomization analysis of VD and risk of thyroid cancer are shown in Supplementary Figure S4.

### Sensitivity analysis results

Cochrane Q test suggested that none of the included SNPs were significantly heterogeneous (Ir: *Q* = 0.12, *p* = 0.73; Ca: *Q* = 187.34, *p* = 0.48; VC: *Q* = 77.14, *p* = 0.11; VD: *Q* = 114.96, *p* = 0.43). Sensitivity analysis by applying the leave-one-out method did not reveal the existence of a single SNP that strongly affected the MR analysis results, indicating that our MR analysis results were robust (Supplementary Figures S9–S12). In addition, the funnel plots were symmetrical, again confirming that our results were reliable (Supplementary Figures S5–S8).

## Discussion

This study analyzed the causal association between Cu, Ir, Zn, Ca, VC, and VD and the risk of TC using a two-sample Mundell randomization method using GWAS data from a public database and the results showed that there was no direct causal relationship.

According to certain research, PTC was favorably correlated with urine Cu levels because Cu is an active oxidation–reduction element that supports thyroid function and lipid metabolism (29). Other research, however, indicated that TSH levels monotonically decline as serum Cu levels rise (30). Co-reduction may enhance thyroid cell oxidative stress, which would inhibit thyroid hormone synthesis and lower levels of thyroid hormone in the blood. Thyroid hormones, on the other hand, may impact blood Cu levels. Our research, however, indicated that there was no clear link between copper and thyroid cancer.

Excess Ir can harm proteins, DNA, and other cellular components (31), and Ir homeostasis is crucial to the biological functions of healthy cells. Studies show that thyroid dysfunction, including hypothyroidism and hyperthyroidism, is associated with hemoglobin levels. Hyperthyroidism is linked to iron deficiency anemia by altering iron metabolism and utilization, increasing iron uptake (32). By changing iron metabolism and use, raising oxidative stress, and accelerating hemolysis, hyperthyroidism is linked to Ir deficient anemia and lowers the RBC survival rate. Hepcidin, a substance secreted by TC cells that can increase intracellular Ir retention and reduce ferroportin (FPN) expression, can encourage the growth of cancer cells. The majority of evidence points to a connection between Ir and TC, albeit the precise mechanism is unknown (8). The increasing concentration of urine Ir quartiles was found to be substantially connected with TC risk, according to Chi Zhang’s study (23). This goes against what we concluded.

Numerous research have been done on zinc and thyroid, and both hypothyroidism and hyperthyroidism are thought to be related to low zinc levels (33). There was a correlation between thyroid volume and Zn content that was positive. Zinc levels and free T3 levels had a statistically significant positive correlation in thyroid-normal patients (34). According to Chi Zhang’s research, Zn is inversely related to thyroid cancer (29). We found no evidence that zinc affected the risk of thyroid cancer. This may be because the initial hypozincemia results from an inflammatory process brought on by the disease (acute phase response), is mostly seen in plasma (but not, for example, erythrocytes), and improves with time in survivors (35).

Ca has a major role in blood clotting and hormone secretion. Abdullah, J’s studies have found that serum Ca ions can serve as a tumor protective factor. However, other studies suggested that there is no significant difference between calcium concentration and thyroid stimulating hormone (TSH; T3, T4) in all groups of different ages, respectively (13).

For VC, there is evidence that the consumption of vitamins with antioxidant properties, such as VC and VD might have some beneficial effects on thyroid-related pathologies (36). F. Karimi’s clinical trial showed that after 3 months of VC, and placebo supplementation patients with AIT received a placebo. In comparison, no statistically significant difference was detected between the two groups in this regard (16). This agrees with what we discovered. There aren’t many findings in the literature about how vitamins may contribute to the development or management of thyroid diseases (37). A randomized controlled trial found that the anti-TgAb and TSH hormone levels in the VD group were much lower than they were at the beginning of the study, indicating that VD supplementation may be effective in reducing disease activity in HT patients (38). The results of our investigation indicate that VD is not related to the emergence of TC. Consequently, additional well-controlled, sizable, longitudinal investigations are required to ascertain whether a connection can be made.

Our study has several advantages. As far as we are aware, this is the first MR investigation to assess the effects of micronutrients on TC. Such a methodology can provide more solid proof of causality between exposure and outcome and can help alleviate constraints brought on by confounding factors frequently present in conventional observational studies. Second, using the largest and most recent GWAS for metabolites allowed for the construction of a strong instrumental variable that was projected to produce unbiased MR estimates. Third, by restricting the study population to those with European ancestry, we were able to lessen the bias brought on by demographic stratification. Fourth, to verify the connections found in the data, we performed several sensitivity analyses (using the weighted median and MR-PRESSO methods, which can minimize the bias brought on by pleiotropic effects).

There are several limitations of our study. First, although we have used MR approaches to guard against confounding, we nevertheless cannot fully exclude residual bias due to unmeasured confounders, which is an established limitation of the MR approach (39). Second, as the number of micronutrient cases in our study was relatively small, we could not completely rule out the possibility that our study may not have adequate power to discover a weak association. Finally, the lack of more detailed subgroup information prevented us from performing further subgroup analyses.

The MR analysis may have identified specific micronutrients that are associated with thyroid cancer risk or outcomes. Future research could focus on conducting mechanistic studies to elucidate the underlying biological mechanisms through which these micronutrients influence thyroid cancer. This could involve *in vitro* experiments, animal models, or molecular studies to explore the pathways and molecular targets involved. Understanding the mechanisms can provide insights into potential therapeutic interventions or preventive strategies.

## Conclusion

Through this large-scale MR study, our analyses attempted to elucidate the potential of micronutrients including Cu, Ir, Zn, Ca, VC, and VD in prophylaxis of thyroid cancer using MR techniques. However, we found little evidence of a causal association between genetically predicted micronutrient concentration on thyroid cancer risk.

## Data availability statement

The original contributions presented in the study are included in the article/Supplementary material, further inquiries can be directed to the corresponding authors.

## Author contributions

JS: Writing – original draft. HZ: Writing – original draft. HJ: Writing – original draft. HL: Writing – original draft. JH: Writing – original draft. SF: Writing – original draft. DY: Writing – review & editing. LY: Writing – review & editing. HT: Writing – review & editing. EL: Writing – review & editing. LL: Writing – review & editing. LC: Writing – original draft.
