# Supplementary material for: The effect of micronutrient on thyroid cancer risk: a Mendelian randomization study
Source: Front Nutr. 2024 Mar 1;11:1331172. doi: 10.3389/fnut.2024.1331172 (PMC10940541; doi:10.3389/fnut.2024.1331172)
Supplement: Supplementary file 1 [file Data_Sheet_1.docx]

Supplementary Material


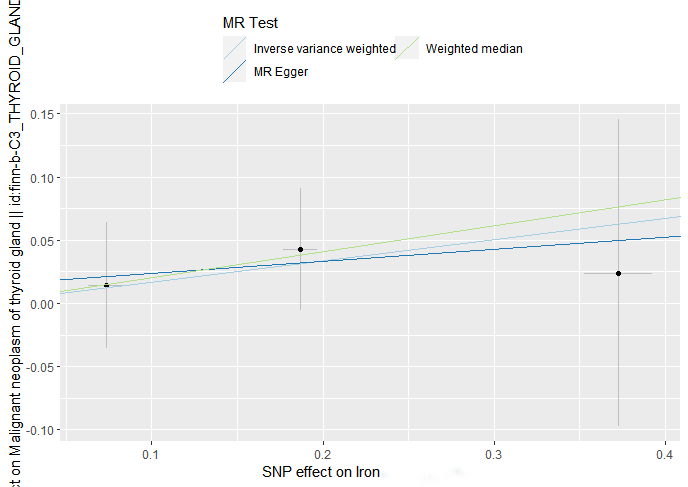


Figure S1 Scatter plot of SNPs associated with Ir and their risk of thyroid cancer.


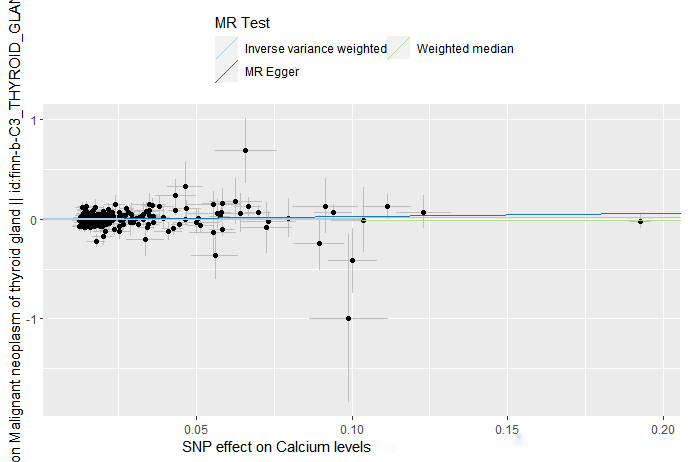


Figure S2 Scatter plot of SNPs associated with Ca and their risk of thyroid cancer.


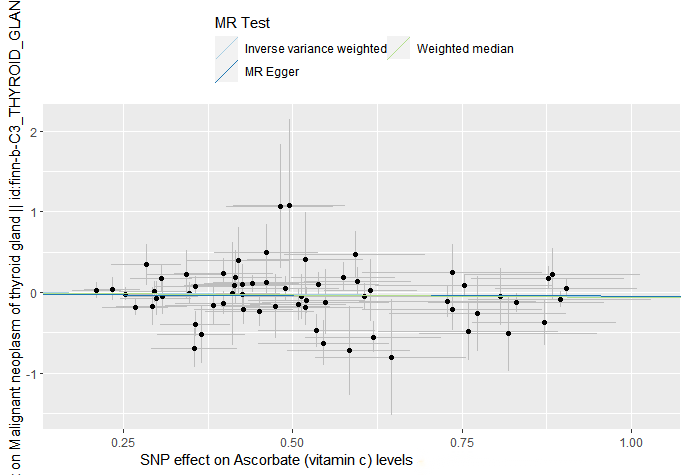
FigureS3 Scatter plot of SNPs associated with VC and their risk of thyroid cancer.
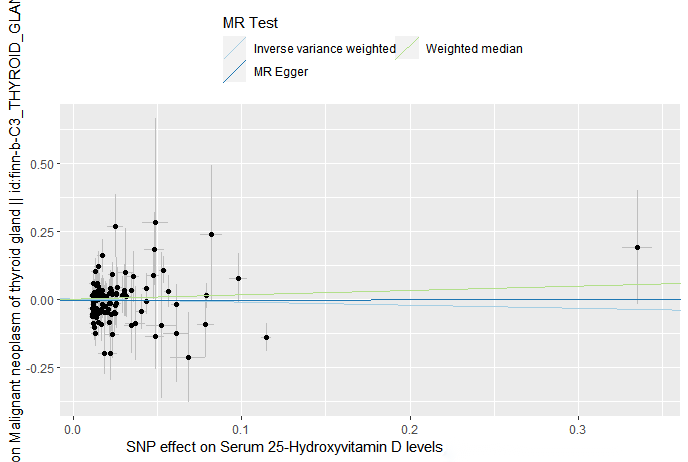
Figure S4 Scatter plot of SNPs associated with VD and their risk of thyroid cancer.


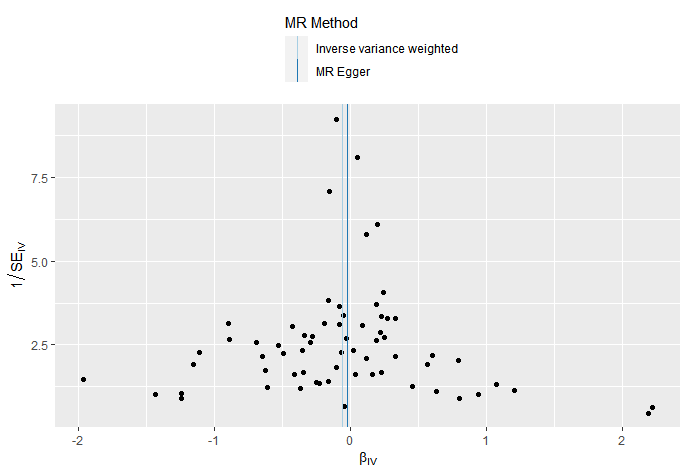


Figure S5 Funnel plot of SNPs associated with VC and their risk of thyroid cancer.


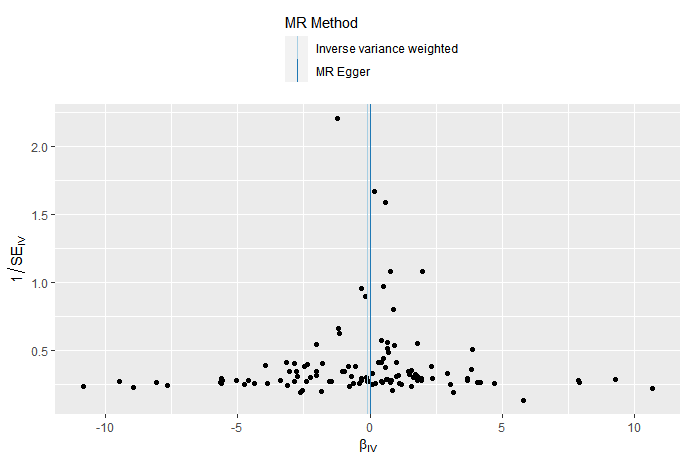


Figure S6 Funnel plot of SNPs associated with VD and their risk of thyroid cancer.


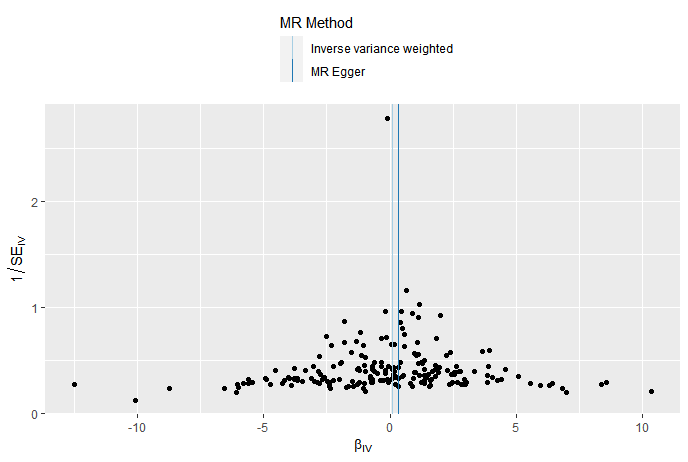


Figure S7 Funnel plot of SNPs associated with Ca and their risk of thyroid cancer.


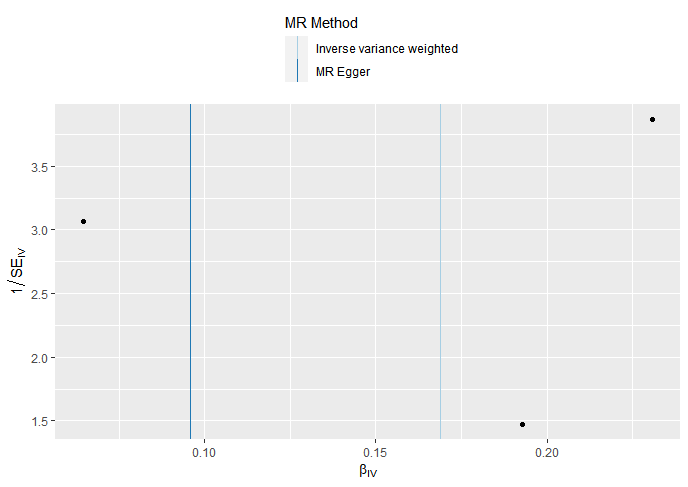
Figure S8 Funnel plot of SNPs associated with Ir and their risk of thyroid cancer.


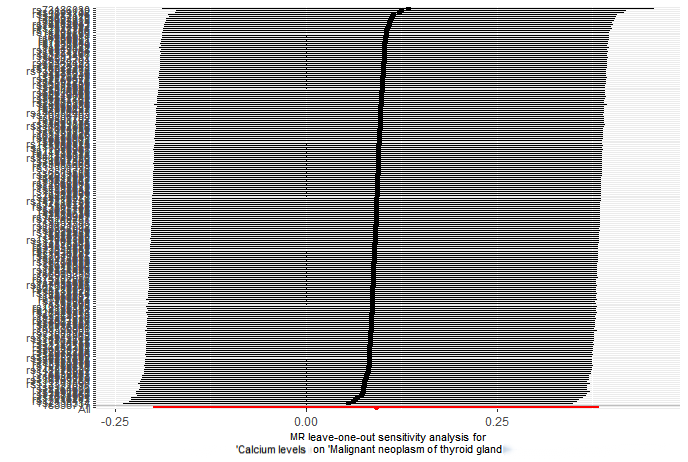


Figure S9 Forest plot of SNPs associated with Ca and their risk of thyroid cancer.


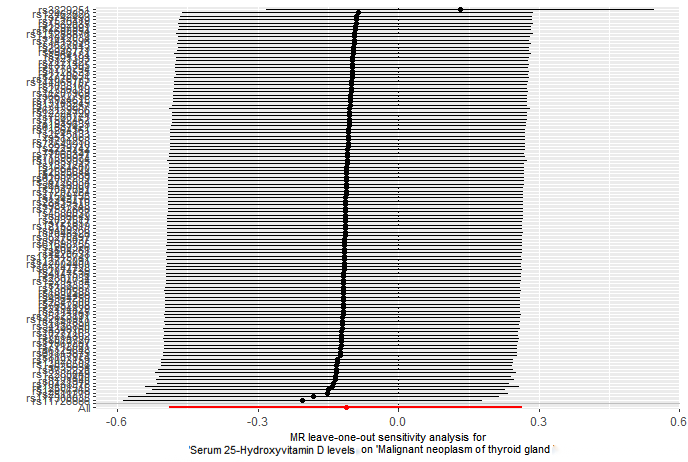


Figure S10 Forest plot of SNPs associated with VD and their risk of thyroid cancer.


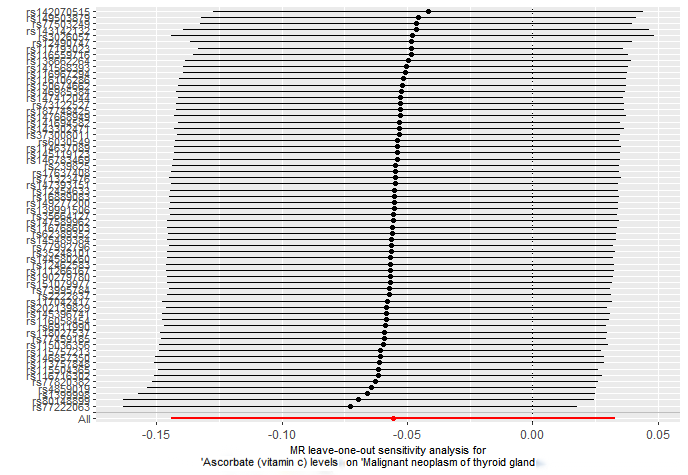
Figure S11 Forest plot of SNPs associated with VC and their risk of thyroid cancer.


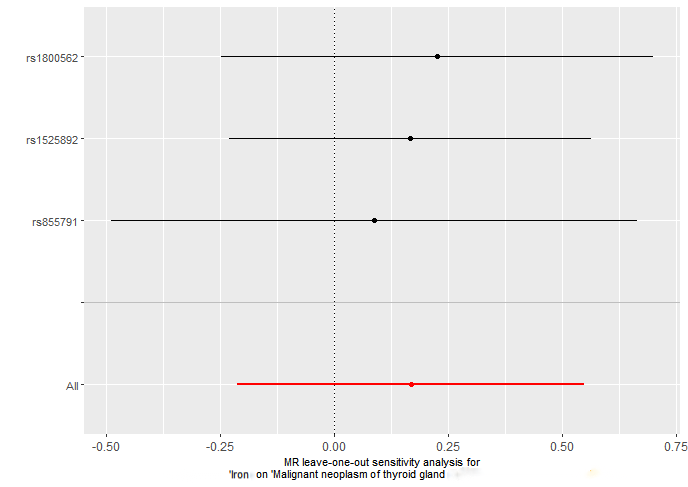


Figure S12 Forest plot of SNPs associated with Ir and their risk of thyroid cancer.

Supplementary Table S 1. 3 SNPs associated with Ir.

| Ir | SNP | chr | pos | beta | se | effect_allele | p | F |
| --- | --- | --- | --- | --- | --- | --- | --- | --- |
|  | rs1525892 | 3 | 133484712 | 0.0736 | 0.0104 | A | 1.65082E-12 | 50.08284024 |
|  | rs1800562 | 6 | 26093141 | 0.3724 | 0.02 | A | 3.95913E-77 | 346.7044 |
|  | rs855791 | 22 | 37462936 | 0.1868 | 0.0101 | G | 4.31221E-77 | 342.0668562 |

Supplementary Table S 2. 173 SNPs associated with Ca.

| Ca | SNP | chr | pos | beta | se | effect_allele | p | F |
| --- | --- | --- | --- | --- | --- | --- | --- | --- |
|  | rs75895430 | 1 | 68268784 | 0.0796 | 0.0071 | G | 5.31E-29 | 125.6925213 |
|  | rs71658797 | 1 | 77967507 | 0.0211 | 0.0038 | A | 2.36E-08 | 30.83171745 |
|  | rs9428344 | 1 | 116816699 | 0.0124 | 0.0022 | T | 2.22E-08 | 31.76859504 |
|  | rs12135382 | 1 | 1098421 | 0.0222 | 0.0026 | T | 1.29E-17 | 72.90532544 |
|  | rs12132412 | 1 | 21820042 | 0.0276 | 0.0024 | G | 8.06E-31 | 132.25 |
|  | rs12411216 | 1 | 155164480 | 0.0254 | 0.0023 | A | 3.87E-28 | 121.9584121 |
|  | rs10917386 | 1 | 23799001 | 0.0192 | 0.0023 | T | 2.36E-16 | 69.68620038 |
|  | rs11204766 | 1 | 151018060 | 0.0411 | 0.0045 | C | 1.30E-19 | 83.41777778 |
|  | rs7546838 | 1 | 156785771 | 0.017 | 0.0024 | G | 1.67E-12 | 50.17361111 |
|  | rs11588907 | 1 | 178509462 | -0.0159 | 0.0026 | T | 1.19E-09 | 37.39792899 |
|  | rs1434282 | 1 | 199010721 | 0.0196 | 0.0025 | T | 8.03E-15 | 61.4656 |
|  | rs116631899 | 1 | 52787887 | 0.0732 | 0.0088 | C | 8.00E-17 | 69.19214876 |
|  | rs697852 | 1 | 226914734 | -0.0207 | 0.0032 | A | 1.65E-10 | 41.84472656 |
|  | rs924204 | 1 | 16513926 | 0.0195 | 0.0023 | G | 7.10E-17 | 71.88090737 |
|  | rs841572 | 1 | 43436051 | 0.0297 | 0.0022 | A | 1.52E-40 | 182.25 |
|  | rs1497826 | 1 | 217471419 | 0.0229 | 0.0024 | G | 3.66E-21 | 91.04340278 |
|  | rs10863512 | 1 | 220075215 | -0.0199 | 0.0023 | T | 2.72E-18 | 74.86011342 |
|  | rs498490 | 10 | 8118677 | -0.0261 | 0.0028 | T | 3.27E-20 | 86.88903061 |
|  | rs112371897 | 10 | 9321880 | 0.0698 | 0.0042 | T | 1.06E-60 | 276.1927438 |
|  | rs12416595 | 10 | 22409964 | 0.0152 | 0.0027 | G | 2.25E-08 | 31.69272977 |
|  | rs17774672 | 10 | 50507709 | -0.0288 | 0.0032 | A | 1.16E-19 | 81 |
|  | rs9415676 | 10 | 65010626 | 0.0147 | 0.0023 | G | 2.59E-10 | 40.84877127 |
|  | rs5786388 | 10 | 80999929 | 0.0188 | 0.0022 | C | 3.93E-17 | 73.02479339 |
|  | rs1061134 | 10 | 100189252 | -0.0216 | 0.0038 | A | 8.71E-09 | 32.31024931 |
|  | rs7086226 | 10 | 22459979 | 0.0191 | 0.0026 | G | 5.31E-13 | 53.96597633 |
|  | rs4082330 | 10 | 65497266 | 0.0208 | 0.0032 | T | 1.12E-10 | 42.25 |
|  | rs11187128 | 10 | 94429708 | -0.0148 | 0.0025 | T | 4.78E-09 | 35.0464 |
|  | rs2419886 | 10 | 115841641 | -0.0192 | 0.0026 | T | 2.99E-13 | 54.53254438 |
|  | rs2762630 | 10 | 9265199 | 0.0317 | 0.0027 | G | 2.83E-31 | 137.8449931 |
|  | rs4935009 | 10 | 52829393 | -0.0193 | 0.003 | C | 2.33E-10 | 41.38777778 |
|  | rs11187838 | 10 | 96038686 | -0.018 | 0.0022 | A | 4.97E-16 | 66.94214876 |
|  | rs4938642 | 11 | 119099906 | 0.0287 | 0.0037 | C | 1.81E-14 | 60.16727538 |
|  | rs73632745 | 11 | 126229617 | -0.0611 | 0.0048 | T | 9.31E-38 | 162.031684 |
|  | rs2004315 | 11 | 13508384 | 0.0315 | 0.0023 | T | 1.93E-42 | 187.5708885 |
|  | rs12793417 | 11 | 34622050 | 0.0172 | 0.0026 | T | 8.03E-11 | 43.76331361 |
|  | rs3841466 | 11 | 77925543 | -0.0158 | 0.0027 | T | 9.86E-09 | 34.2441701 |
|  | rs7108820 | 11 | 101999974 | 0.0143 | 0.0022 | C | 1.98E-10 | 42.25 |
|  | rs2583435 | 11 | 2958818 | -0.0233 | 0.0024 | C | 2.96E-22 | 94.25173611 |
|  | rs144562710 | 11 | 47933609 | 0.02 | 0.0026 | A | 1.37E-14 | 59.17159763 |
|  | rs4517550 | 11 | 71521071 | 0.0146 | 0.0023 | C | 3.91E-10 | 40.29489603 |
|  | rs302655 | 11 | 87891387 | -0.0186 | 0.0023 | T | 1.56E-16 | 65.39886578 |
|  | rs11228382 | 11 | 68617316 | -0.0182 | 0.0022 | T | 5.67E-16 | 68.43801653 |
|  | rs1182922 | 11 | 118932859 | -0.0134 | 0.0023 | A | 5.13E-09 | 33.94328922 |
|  | rs949300 | 11 | 122553139 | 0.0146 | 0.0023 | A | 1.20E-10 | 40.29489603 |
|  | rs775249 | 12 | 57977003 | -0.0155 | 0.0025 | T | 4.00E-10 | 38.44 |
|  | rs3026445 | 12 | 110723203 | -0.0172 | 0.0025 | C | 1.45E-11 | 47.3344 |
|  | rs17884869 | 12 | 123519112 | -0.1113 | 0.0079 | A | 1.05E-44 | 198.488864 |
|  | rs73202933 | 12 | 90213083 | 0.0235 | 0.0033 | C | 9.88E-13 | 50.71166208 |
|  | rs117213754 | 12 | 4006794 | 0.1036 | 0.0102 | A | 3.05E-24 | 103.1618608 |
|  | rs117080167 | 12 | 12205320 | -0.0318 | 0.0045 | T | 2.35E-12 | 49.93777778 |
|  | rs7964801 | 12 | 49097212 | -0.0271 | 0.0026 | T | 1.43E-24 | 108.6405325 |
|  | rs6580981 | 12 | 54723028 | -0.0152 | 0.0023 | A | 1.29E-11 | 43.67485822 |
|  | rs7323058 | 13 | 42548503 | -0.0348 | 0.0034 | T | 7.06E-24 | 104.7612457 |
|  | rs9524868 | 13 | 95909661 | -0.0135 | 0.0024 | T | 1.15E-08 | 31.640625 |
|  | rs2249825 | 13 | 31037903 | -0.0165 | 0.0026 | C | 2.55E-10 | 40.27366864 |
|  | rs1577452 | 13 | 110497532 | -0.0212 | 0.0025 | G | 4.88E-17 | 71.9104 |
|  | rs1023229 | 13 | 20270925 | -0.02 | 0.0034 | A | 7.21E-09 | 34.60207612 |
|  | rs35852840 | 14 | 64595763 | 0.029 | 0.0053 | A | 4.30E-08 | 29.93948024 |
|  | rs17580 | 14 | 94847262 | 0.0466 | 0.0058 | A | 5.51E-16 | 64.5529132 |
|  | rs58087925 | 14 | 105983096 | -0.0215 | 0.0029 | T | 2.83E-13 | 54.96432818 |
|  | rs28693943 | 14 | 60640916 | -0.0192 | 0.0029 | T | 7.66E-11 | 43.83353151 |
|  | rs28929474 | 14 | 94844947 | 0.1231 | 0.0088 | T | 2.20E-44 | 195.6819473 |
|  | rs11621792 | 14 | 24871926 | 0.0156 | 0.0024 | T | 1.54E-10 | 42.25 |
|  | rs7144433 | 14 | 90850229 | -0.0252 | 0.0033 | T | 4.76E-14 | 58.31404959 |
|  | rs11629876 | 15 | 96666402 | -0.0157 | 0.0024 | T | 8.21E-11 | 42.79340278 |
|  | rs4324076 | 15 | 51510868 | -0.0201 | 0.0022 | C | 9.14E-20 | 83.4731405 |
|  | rs60616569 | 15 | 78271261 | -0.0178 | 0.0028 | A | 1.70E-10 | 40.41326531 |
|  | rs2047824 | 15 | 69609456 | -0.0254 | 0.0023 | C | 5.25E-29 | 121.9584121 |
|  | rs139974673 | 15 | 44027885 | 0.1002 | 0.0079 | C | 1.58E-36 | 160.8722961 |
|  | rs41278174 | 16 | 16259596 | 0.0501 | 0.0076 | A | 4.26E-11 | 43.4558518 |
|  | rs34042070 | 16 | 72101525 | 0.0167 | 0.0027 | G | 3.80E-10 | 38.25651578 |
|  | rs12933858 | 16 | 81566121 | 0.0173 | 0.0023 | T | 5.55E-14 | 56.57655955 |

Supplementary Table S 3. 118 SNPs associated with VD.

| **VD** | ***SNP*** | ***chr*** | ***pos*** | ***beta*** | ***se*** | ***effect_allele*** | ***p*** | ***F*** |
| --- | --- | --- | --- | --- | --- | --- | --- | --- |
|  | rs11207969 | 1 | 62911751 | 0.0209396 | 0.00212672 | G | 7.14E-23 | 96.94292785 |
|  | rs11264361 | 1 | 155289545 | 0.0174875 | 0.00234081 | G | 7.97E-14 | 55.811427 |
|  | rs61747728 | 1 | 179526214 | 0.0303061 | 0.00526894 | T | 8.83E-09 | 33.08366371 |
|  | rs2807834 | 1 | 220970593 | -0.0150625 | 0.00218678 | G | 5.66E-12 | 47.44428798 |
|  | rs512083 | 1 | 46027355 | 0.0122172 | 0.00204286 | C | 2.23E-09 | 35.76565288 |
|  | rs6672758 | 1 | 230303512 | 0.0162478 | 0.0025554 | T | 2.04E-10 | 40.42698452 |
|  | rs2494429 | 1 | 2339395 | -0.0148459 | 0.00267333 | G | 2.80E-08 | 30.83954186 |
|  | rs1343776 | 1 | 41757718 | 0.0180762 | 0.00245028 | A | 1.62E-13 | 54.42304602 |
|  | rs7528419 | 1 | 109817192 | 0.0215389 | 0.00243165 | G | 8.17E-19 | 78.45938691 |
|  | rs115288876 | 1 | 152000117 | 0.0788065 | 0.00498229 | A | 2.36E-56 | 250.187769 |
|  | rs35823191 | 1 | 17560123 | -0.0232636 | 0.00214072 | C | 1.65E-27 | 118.0957071 |
|  | rs61813875 | 1 | 152536650 | 0.0821291 | 0.00658876 | G | 1.16E-35 | 155.3770067 |
|  | rs2398113 | 10 | 10076429 | -0.0117606 | 0.00205797 | G | 1.10E-08 | 32.65734536 |
|  | rs12775091 | 10 | 91524012 | 0.0155618 | 0.00247693 | T | 3.33E-10 | 39.47227814 |
|  | rs2297991 | 10 | 113913222 | 0.0127547 | 0.00225578 | C | 1.57E-08 | 31.97032216 |
|  | rs77532868 | 10 | 88081438 | 0.0259557 | 0.00456301 | T | 1.28E-08 | 32.35658291 |
|  | rs144965707 | 11 | 14059511 | -0.0348143 | 0.00421754 | A | 1.52E-16 | 68.1391818 |
|  | rs1627043 | 11 | 71110175 | -0.0486441 | 0.00566106 | C | 8.49E-18 | 73.83543357 |
|  | rs2847500 | 11 | 120114421 | -0.022548 | 0.00308665 | A | 2.77E-13 | 53.36312795 |
|  | rs17473257 | 11 | 14283186 | -0.0611372 | 0.00780044 | A | 4.59E-15 | 61.42892117 |
|  | rs117300835 | 11 | 15118975 | -0.334985 | 0.00886005 | A | 1.00E-200 | 1429.480962 |
|  | rs2511279 | 11 | 71130419 | 0.0981721 | 0.00520826 | G | 2.98E-79 | 355.2964346 |
|  | rs3829251 | 11 | 71194559 | -0.114453 | 0.00298056 | A | 1.00E-200 | 1474.547013 |
|  | rs11023159 | 11 | 14262063 | 0.0482117 | 0.00572507 | C | 3.73E-17 | 70.91584607 |
|  | rs733454 | 11 | 76477721 | 0.0188545 | 0.00340001 | T | 2.93E-08 | 30.75173695 |
|  | rs111515741 | 11 | 14370944 | -0.0487364 | 0.00779044 | A | 3.95E-10 | 39.13658418 |
|  | rs12283049 | 11 | 14690192 | -0.0564566 | 0.00240614 | G | 9.62E-122 | 550.5384478 |
|  | rs11600054 | 11 | 14690511 | 0.0681747 | 0.0101478 | A | 1.84E-11 | 45.13388028 |
|  | rs964184 | 11 | 116648917 | 0.0406845 | 0.00298936 | C | 3.50E-42 | 185.2258208 |
|  | rs61887421 | 11 | 70949673 | -0.036726 | 0.00597754 | C | 8.05E-10 | 37.74872416 |
|  | rs7955128 | 12 | 38684121 | 0.0130617 | 0.00203856 | T | 1.48E-10 | 41.05371024 |
|  | rs1038165 | 12 | 68665940 | 0.0115149 | 0.00205629 | T | 2.15E-08 | 31.35823529 |
|  | rs73413596 | 12 | 111582630 | 0.0223468 | 0.00388928 | C | 9.15E-09 | 33.01355277 |
|  | rs28435470 | 12 | 133067473 | -0.0118696 | 0.00214831 | A | 3.29E-08 | 30.52658814 |
|  | rs57601828 | 12 | 93192127 | 0.011542 | 0.00208192 | T | 2.96E-08 | 30.73506001 |
|  | rs1871395 | 12 | 21352315 | -0.0203733 | 0.00282689 | G | 5.72E-13 | 51.94035834 |
|  | rs2171427 | 12 | 24822154 | -0.0165489 | 0.00281738 | A | 4.26E-09 | 34.50224928 |
|  | rs10859995 | 12 | 96375682 | -0.0436264 | 0.00205452 | C | 4.60E-100 | 450.8976869 |
|  | rs4580037 | 13 | 55702646 | -0.0135627 | 0.00225079 | C | 1.68E-09 | 36.30967461 |
|  | rs8018720 | 14 | 39556185 | -0.0344962 | 0.00266074 | C | 1.94E-38 | 168.0883589 |
|  | rs2756119 | 14 | 104001517 | 0.0121434 | 0.0021104 | A | 8.71E-09 | 33.10937976 |
|  | rs142004400 | 14 | 50829560 | -0.0310034 | 0.00559561 | C | 3.01E-08 | 30.69896769 |
|  | rs1532085 | 15 | 58683366 | 0.0252805 | 0.00208639 | G | 8.60E-34 | 146.8183481 |
|  | rs1800588 | 15 | 58723675 | -0.0305021 | 0.00246932 | T | 4.73E-35 | 152.5825024 |
|  | rs62007299 | 15 | 77711719 | -0.0124205 | 0.00224413 | A | 3.12E-08 | 30.63247835 |
|  | rs325393 | 15 | 100229260 | -0.0136497 | 0.00227676 | T | 2.03E-09 | 35.94278352 |
|  | rs12324720 | 15 | 64092140 | -0.0149159 | 0.00267451 | A | 2.45E-08 | 31.10358681 |
|  | rs1684600 | 16 | 4594671 | -0.0125301 | 0.00221719 | T | 1.59E-08 | 31.93767166 |
|  | rs11542462 | 16 | 82033810 | -0.0247803 | 0.00298265 | A | 9.72E-17 | 69.02533638 |
|  | rs77924615 | 16 | 20392332 | -0.0152475 | 0.00259019 | A | 3.94E-09 | 34.65245748 |
|  | rs11076175 | 16 | 57006378 | 0.0229033 | 0.00266997 | G | 9.64E-18 | 73.58399458 |
|  | rs11867297 | 17 | 66433493 | 0.0135432 | 0.00209454 | T | 1.01E-10 | 41.80856551 |
|  | rs61698755 | 17 | 79257880 | -0.011465 | 0.0020505 | C | 2.25E-08 | 31.26285029 |
|  | rs9946771 | 18 | 28918628 | -0.0233992 | 0.00407655 | T | 9.47E-09 | 32.94704523 |
|  | rs2037511 | 18 | 61366207 | 0.0176624 | 0.00272732 | A | 9.41E-11 | 41.93988521 |
|  | rs77960347 | 18 | 47109955 | -0.0525688 | 0.00905963 | G | 6.53E-09 | 33.6693866 |
|  | rs10438978 | 18 | 47158186 | -0.0172243 | 0.00264437 | C | 7.34E-11 | 42.42664784 |
|  | rs1048328 | 19 | 51527364 | 0.0313497 | 0.00374376 | A | 5.58E-17 | 70.12143237 |
|  | rs142158911 | 19 | 11190534 | 0.026284 | 0.00323446 | A | 4.43E-16 | 66.03578543 |
|  | rs12462826 | 19 | 11955767 | -0.0132119 | 0.0021149 | A | 4.18E-10 | 39.02571614 |
|  | rs4420638 | 19 | 45422946 | -0.0192973 | 0.00265905 | G | 3.95E-13 | 52.66718295 |
|  | rs8107974 | 19 | 19388500 | 0.0355672 | 0.00382316 | T | 1.36E-20 | 86.54747593 |
|  | rs62129966 | 19 | 48374950 | 0.0611636 | 0.00276373 | A | 1.60E-108 | 489.7730152 |
|  | rs1042034 | 2 | 21225281 | -0.0151254 | 0.00250017 | T | 1.45E-09 | 36.59945833 |
|  | rs1260326 | 2 | 27730940 | 0.0197194 | 0.00207421 | C | 1.96E-21 | 90.38199967 |
|  | rs35270497 | 2 | 38259872 | 0.0156723 | 0.0026815 | T | 5.08E-09 | 34.15937139 |
|  | rs7569755 | 2 | 118648261 | 0.0136395 | 0.00225626 | A | 1.49E-09 | 36.54421288 |
|  | rs2710651 | 2 | 63166379 | -0.0115892 | 0.00203474 | A | 1.23E-08 | 32.44061434 |
|  | rs3732220 | 2 | 234627048 | -0.0478406 | 0.00363266 | A | 1.31E-39 | 173.4377904 |
|  | rs727857 | 2 | 58981967 | -0.0120548 | 0.00209882 | A | 9.27E-09 | 32.98903646 |
|  | rs7580771 | 2 | 101428119 | -0.0165625 | 0.0026652 | T | 5.15E-10 | 38.61821298 |
|  | rs1047891 | 2 | 211540507 | -0.0133984 | 0.00218016 | A | 7.96E-10 | 37.76844843 |

Supplementary Table S4. 68SNPs associated with VC.

| VC | SNP | chr | pos | beta | se | effect_allele | p | F |
| --- | --- | --- | --- | --- | --- | --- | --- | --- |
|  | rs147412044 | 1 | 167758303 | -0.8188 | 0.1311 | T | 4.26E-10 | 39.00769468 |
|  | rs115757213 | 1 | 211685549 | -0.3982 | 0.0725 | G | 3.93E-08 | 30.16660927 |
|  | rs145396741 | 1 | 225599808 | -0.3569 | 0.0611 | A | 5.20E-09 | 34.12012986 |
|  | rs77503249 | 1 | 234790766 | -0.3558 | 0.052 | A | 7.92E-12 | 46.81717456 |
|  | rs35248101 | 10 | 13171617 | -0.2336 | 0.0409 | C | 1.16E-08 | 32.6211345 |
|  | rs138662264 | 10 | 101464483 | -0.8716 | 0.1062 | C | 2.31E-16 | 67.35741468 |
|  | rs141694582 | 10 | 30833134 | -0.3648 | 0.0649 | A | 1.93E-08 | 31.59513866 |
|  | rs77992796 | 10 | 129203425 | -0.4948 | 0.0831 | A | 2.66E-09 | 35.45335459 |
|  | rs3026057 | 10 | 119014127 | -0.8949 | 0.1352 | A | 3.60E-11 | 43.81225765 |
|  | rs118027537 | 12 | 16085254 | -0.8831 | 0.1303 | C | 1.22E-11 | 45.93364801 |
|  | rs77459185 | 12 | 77206531 | -0.3061 | 0.0454 | G | 1.54E-11 | 45.45848454 |
|  | rs77820382 | 12 | 111511225 | -0.5747 | 0.0767 | A | 6.84E-14 | 56.14248949 |
|  | rs145119123 | 13 | 108479750 | -0.2923 | 0.0526 | T | 2.81E-08 | 30.88062933 |
|  | rs117042417 | 13 | 66201226 | -0.4897 | 0.0789 | A | 5.34E-10 | 38.52176714 |
|  | rs142070515 | 13 | 92501248 | -0.6196 | 0.1003 | G | 6.49E-10 | 38.16110591 |
|  | rs146783469 | 14 | 60859899 | -0.7732 | 0.1311 | G | 3.64E-09 | 34.78390268 |
|  | rs143302471 | 16 | 2895094 | -0.5206 | 0.086 | A | 1.39E-09 | 36.64472147 |
|  | rs141568393 | 16 | 22944021 | -0.4512 | 0.0805 | G | 2.08E-08 | 31.41567686 |
|  | rs373008011 | 16 | 84127498 | -0.5831 | 0.0904 | T | 1.09E-10 | 41.60535403 |
|  | rs17637408 | 16 | 26295044 | -0.2981 | 0.0527 | C | 1.49E-08 | 31.99651819 |
|  | rs1399998 | 16 | 27163332 | -0.753 | 0.1307 | C | 8.36E-09 | 33.1924093 |
|  | rs117193023 | 18 | 5643874 | -0.355 | 0.0636 | T | 2.35E-08 | 31.15605593 |
|  | rs80148899 | 18 | 22668598 | -0.9042 | 0.1051 | T | 7.71E-18 | 74.01565271 |
|  | rs12454633 | 18 | 40494893 | -0.3071 | 0.0532 | A | 7.89E-09 | 33.32240729 |
|  | rs12462583 | 19 | 38905792 | -0.2106 | 0.0358 | A | 4.24E-09 | 34.60594239 |
|  | rs116716302 | 2 | 140681452 | -0.461 | 0.0784 | T | 4.15E-09 | 34.57560001 |
|  | rs146985384 | 2 | 141602409 | -0.7361 | 0.1332 | G | 3.25E-08 | 30.53972948 |
|  | rs147589962 | 2 | 185869804 | -0.426 | 0.0706 | C | 1.62E-09 | 36.40908763 |
|  | rs114637089 | 2 | 48801442 | -0.3821 | 0.0696 | A | 4.03E-08 | 30.13946897 |
|  | rs73995784 | 2 | 235971015 | -0.4812 | 0.0785 | G | 8.70E-10 | 37.57611911 |
|  | rs150674662 | 2 | 122911880 | -0.3976 | 0.0704 | A | 1.60E-08 | 31.89682335 |
|  | rs6030549 | 20 | 41519220 | -0.645 | 0.1114 | C | 7.00E-09 | 33.5234763 |
|  | rs187748425 | 21 | 40558118 | -0.5082 | 0.071 | A | 8.40E-13 | 51.23333466 |
|  | rs147668949 | 21 | 28314693 | -0.7291 | 0.131 | A | 2.61E-08 | 30.97644718 |
|  | rs149503879 | 21 | 45271053 | -0.5354 | 0.0866 | A | 6.31E-10 | 38.22266373 |
|  | rs113757848 | 22 | 34545881 | -0.538 | 0.0897 | A | 2.01E-09 | 35.97324912 |
|  | rs12490747 | 3 | 9092890 | -0.2677 | 0.0484 | A | 3.19E-08 | 30.59186958 |
|  | rs71323476 | 3 | 116616727 | -0.6054 | 0.1104 | T | 4.13E-08 | 30.07091801 |
|  | rs116768603 | 3 | 1348020 | -0.3479 | 0.0507 | G | 7.14E-12 | 47.08612366 |
|  | rs4859019 | 3 | 88440502 | -0.4399 | 0.0701 | C | 3.44E-10 | 39.37965328 |
|  | rs115504365 | 3 | 136506946 | -0.5922 | 0.1041 | A | 1.26E-08 | 32.36199952 |
|  | rs2222837 | 3 | 160331821 | -0.4193 | 0.0769 | A | 4.88E-08 | 29.73014622 |
|  | rs115036356 | 3 | 13191877 | -0.4144 | 0.0744 | A | 2.55E-08 | 31.02370216 |
|  | rs149277200 | 3 | 184405033 | -0.5132 | 0.0861 | G | 2.53E-09 | 35.52769178 |
|  | rs141109644 | 3 | 196034184 | -0.3335 | 0.0604 | G | 3.39E-08 | 30.48721821 |
|  | rs73122527 | 4 | 27134001 | -0.5183 | 0.0863 | A | 1.94E-09 | 36.06955848 |
|  | rs151079977 | 4 | 95995355 | -0.3436 | 0.0593 | G | 6.76E-09 | 33.5735236 |
|  | rs116058454 | 4 | 82799684 | -0.7361 | 0.1332 | A | 3.25E-08 | 30.53972948 |
|  | rs147393151 | 4 | 138234060 | -0.5488 | 0.0938 | T | 4.91E-09 | 34.2312319 |
|  | rs202139829 | 4 | 144621585 | -0.2837 | 0.052 | A | 4.90E-08 | 29.7654179 |
|  | rs62389352 | 5 | 163304133 | -0.6152 | 0.0682 | T | 1.95E-19 | 81.36992286 |
|  | rs111266167 | 5 | 132512946 | -0.4258 | 0.0761 | T | 2.25E-08 | 31.30703946 |
|  | rs145489384 | 5 | 92700914 | -0.2956 | 0.0523 | G | 1.59E-08 | 31.94519046 |
|  | rs116106286 | 5 | 172207791 | -0.4276 | 0.0727 | A | 4.08E-09 | 34.59446123 |
|  | rs6911990 | 6 | 152669178 | -0.4612 | 0.0768 | C | 1.92E-09 | 36.06252713 |
|  | rs16889083 | 6 | 36729068 | -0.2534 | 0.0396 | T | 1.52E-10 | 40.94707173 |
|  | rs239825 | 6 | 54773200 | -0.4749 | 0.08 | C | 2.95E-09 | 35.23906406 |
|  | rs146857350 | 6 | 112518469 | -0.5956 | 0.079 | A | 4.77E-14 | 56.84014741 |
|  | rs116967294 | 6 | 95056239 | -0.7594 | 0.1334 | C | 1.26E-08 | 32.40630584 |
|  | rs76715584 | 6 | 152513466 | -0.2278 | 0.0379 | T | 1.86E-09 | 36.12676047 |
|  | rs144580260 | 7 | 48052891 | -0.519 | 0.0859 | A | 1.54E-09 | 36.50466674 |
|  | rs143142132 | 7 | 12082686 | -0.8303 | 0.1305 | T | 1.96E-10 | 40.48079682 |
|  | rs181246816 | 8 | 1278957 | -0.2302 | 0.0398 | T | 7.38E-09 | 33.45372592 |
|  | rs77222063 | 8 | 21773577 | -0.8769 | 0.1134 | G | 1.02E-14 | 59.7962613 |
|  | rs35664127 | 9 | 73622580 | -0.411 | 0.0702 | A | 4.90E-09 | 34.2775221 |
|  | rs139991506 | 9 | 23704505 | -0.8069 | 0.1311 | G | 7.42E-10 | 37.88210012 |
|  | rs190279780 | 9 | 8949161 | -0.416 | 0.0687 | A | 1.44E-09 | 36.66681498 |
|  | rs116559716 | 9 | 98210170 | -0.5459 | 0.0968 | A | 1.69E-08 | 31.80354078 |

Supplementary Table S 5. 2 SNPs associated with Zn.

| Zn | SNP | chr | pos | beta | se | effect_allele | p | F |
| --- | --- | --- | --- | --- | --- | --- | --- | --- |
|  | rs1532423 | 8 | 86268313 | -0.178 | 0.026 | G | 6.40E-12 | 46.86982249 |
|  | rs2120019 | 15 | 75334184 | -0.287 | 0.033 | C | 1.55E-18 | 75.63728191 |

Supplementary Table S 6. 2 SNPs associated with Cu.

| Cu | SNP | chr | pos | beta | se | effect_allele | p | F |
| --- | --- | --- | --- | --- | --- | --- | --- | --- |
|  | rs1175550 | 1 | 3691528 | 0.198 | 0.032 | G | 5.03002E-10 | 38.28515625 |
|  | rs2769264 | 1 | 151344741 | 0.313 | 0.034 | G | 2.63027E-20 | 84.7482699 |
